# Hepatocyte-Derived Prostaglandin E2-Modulated Macrophage M1-Type Polarization via mTOR-NPC1 Axis-Regulated Cholesterol Transport from Lysosomes to the Endoplasmic Reticulum in Hepatitis B Virus x Protein-Related Nonalcoholic Steatohepatitis

**DOI:** 10.3390/ijms231911660

**Published:** 2022-10-01

**Authors:** You Lan, Bo Qian, Hai-Yan Huang, Pan Wang, Ting Li, Qi Yuan, Han-Yu Zhang, Yu-Chun Lin, Zhong-Ning Lin

**Affiliations:** State Key Laboratory of Molecular Vaccinology and Molecular Diagnostics, School of Public Health, Xiamen University, Xiamen, 361102, China

**Keywords:** hepatitis B virus x protein, prostaglandin E2, lysosomal mTOR-NPC1 signal axis, cholesterol transport, endoplasmic reticulum stress, macrophage M1-type polarization, nonalcoholic steatohepatitis

## Abstract

Lipid metabolic dysregulation and liver inflammation have been reported to be associated with nonalcoholic steatohepatitis (NASH), but the underlying mechanisms remain unclear. Hepatitis B virus x protein (HBx) is a risk factor for NASH. Based on metabolomic and transcriptomic screens and public database analysis, we found that HBx-expressing hepatocyte-derived prostaglandin E2 (PGE2) induced macrophage polarization imbalance via prostaglandin E2 receptor 4 (EP4) through in vitro, ex vivo, and in vivo models. Here, we revealed that the M1-type polarization of macrophages induced by endoplasmic reticulum oxidoreductase-1-like protein α (ERO1α)-dependent endoplasmic reticulum stress was associated with the HBx-related hepatic NASH phenotype. Mechanistically, HBx promoted Niemann–Pick type C1 (NPC1)/oxysterol-binding protein-related protein 5 (ORP5)-mediated cholesterol transport from the lysosome to the endoplasmic reticulum via mammalian target of rapamycin (mTOR) activation. This study provides a novel basis for screening potential biomarkers in the macrophage mTOR–cholesterol homeostasis–polarization regulatory signaling pathway and evaluating targeted interventions for HBx-associated NASH.

## 1. Introduction

Nonalcoholic fatty liver disease (NAFLD) has become an emerging public health problem, with a global prevalence of 25%, including a range of liver diseases, from fatty liver to nonalcoholic steatohepatitis (NASH) [1,2]. NASH is characterized by lipid accumulation and inflammatory damage following lipid metabolism disorders [3,4]. Regional immune regulation and immuno-metabolic mechanisms in the liver are the current frontiers and hot spots in the study of NASH. Metabolic perturbations affect the immune cell phenotype and function, disrupting hepatic homeostasis and promoting the development of NASH [5,6]. The increasing number of cases of hepatitis B and hepatic steatosis indicates an association between the hepatitis B virus (HBV) and NAFLD [7]. Additionally, studies have suggested that hepatitis B virus x protein (HBx) is a possible risk factor for a fatty liver. Wu et al. found that HBx induces hepatic steatosis by enhancing the expression of liver fatty acid binding protein [8]. We also found that HBx triggers hepatic steatosis via the induction of cyclooxygenase 2 (COX-2) in hepatocytes [9]. COX-2 catalyzes the synthesis of prostaglandin E2 (PGE2), an inflammatory lipid mediator, from arachidonic acid [10]. Su et al. found that senescent preosteoclasts promote metabolic syndrome associated with osteoarthritis through the COX-2/PGE2 axis [11]. PGE2 stimulates different immune cells, such as macrophages, dendritic cells, neutrophils, and T cells, to exert various biological effects through PGE2 receptors (EP1-EP4) [12]. The specific role and related mechanism of PGE2 in HBV/HBx-related NASH need further exploration.

Lipotoxicity induced by cholesterol accumulation within macrophages induces the formation of foam cells with an M1-type polarization and accelerates the transition of NAFLD from simple steatosis to NASH [13,14]. Macrophages obtain cholesterol primarily through the uptake of low-density lipoprotein (LDL) from the surrounding environment [15]. LDL then enters the lysosomes (Lyso) and is degraded to free cholesterol (FC) by lysosomal acid lipase (LAL), and through feedback inhibition, the increase in intracellular FC levels can prevent LDL receptors from reducing cholesterol uptake [16]. Unlike LDL, oxidized low-density lipoprotein (ox-LDL) accumulates in the lysosome, and intracellular FC levels do not negatively affect scavenger receptors, leading to cholesterol accumulation in macrophages [17]. As a result, excess cholesterol is transferred from the lysosome to other organelles and causes stress and dysfunction [18]. In atherosclerotic disease, cholesterol accumulation in the endoplasmic reticulum (ER) can trigger ER stress through PERK, IRE1α, and ATF6 pathways, which can launch proinflammatory responses, reduce cholesterol efflux, or/and increase cholesterol uptake, which can lead to foam cell formation via lipid droplet accumulation [19]. Our previous studies found that ER stress mediated the inflammatory response triggered by ultra-small superparamagnetic iron oxide nanoparticles [20]; HBx induced cell-cycle arrest and apoptosis via ER stress [21]; and aflatoxin B1 induced COX-2 upregulation and inflammasome activation via the modulation of endoplasmic reticulum oxidoreductase-1-like protein α (ERO1α)-dependent ER redox homeostasis and ER stress [22]. Therefore, we hypothesized that cholesterol accumulation-induced ER stress is also involved in the proinflammatory M1-type polarization of macrophages, contributing to the development of HBx-associated NASH.

Cholesterol transport between organelles is mainly divided into vesicular and non-vesicular forms [23]. Recent studies have proposed that inter-organelle membrane contact sites (MCS), as non-vesicular lipid transfer hubs, are central to the metabolic integration and signal transduction between organelles by facilitating the exchange of small molecules, such as cholesterol, in local regions through tightly arranged bundle complexes [24]. For example, Höglinger et al. found that Niemann–Pick type C1 (NPC1) regulates endocytic organelle–ER contact to mediate lipoprotein-derived cholesterol transport from the lysosome to the ER [25]. In addition, cholesterol transporters bridging MCS between the lysosome and ER include oxysterol-binding protein (OSBP)-related proteins (ORPs) [26], steroidogenic acute regulatory protein-related lipid transfer domain (START) [27], GramD [25], and SLC38A9 [28]. Notably, the mammalian target of rapamycin (mTOR) response to various environmental cues, from cytokine stimulation to nutrients to stress, may be associated with cholesterol transport [29]. In a previous study, we found that COX-2 modulated Ca^2+^ transport across the mitochondria-associated ER membrane (MAM) to mediate superparamagnetic iron oxide nanoparticle-induced hepatotoxicity [30]. Therefore, we hypothesized that Lyso-ER cholesterol transport induces ER stress and is regulated by mTOR signaling.

We constructed in vitro, ex vivo, and in vivo experimental models of HBx-expressing hepatocytes to activate macrophages in the current study. We found that PGE2-EP4-mTOR signaling promotes Lyso-ER cholesterol transport via NPC1 and aggravates ER stress-induced macrophage M1-type polarization through the ERO1α-mediated disturbance of ER redox in HBx-related NASH. EP4 may be a potential targeted intervention for the occurrence and progression of NASH induced by HBx.

## 2. Results

### 2.1. PGE2-EP4 Induced ER Stress-Mediated Macrophage Polarization Imbalance via mTOR Signaling

**Figure 1:**
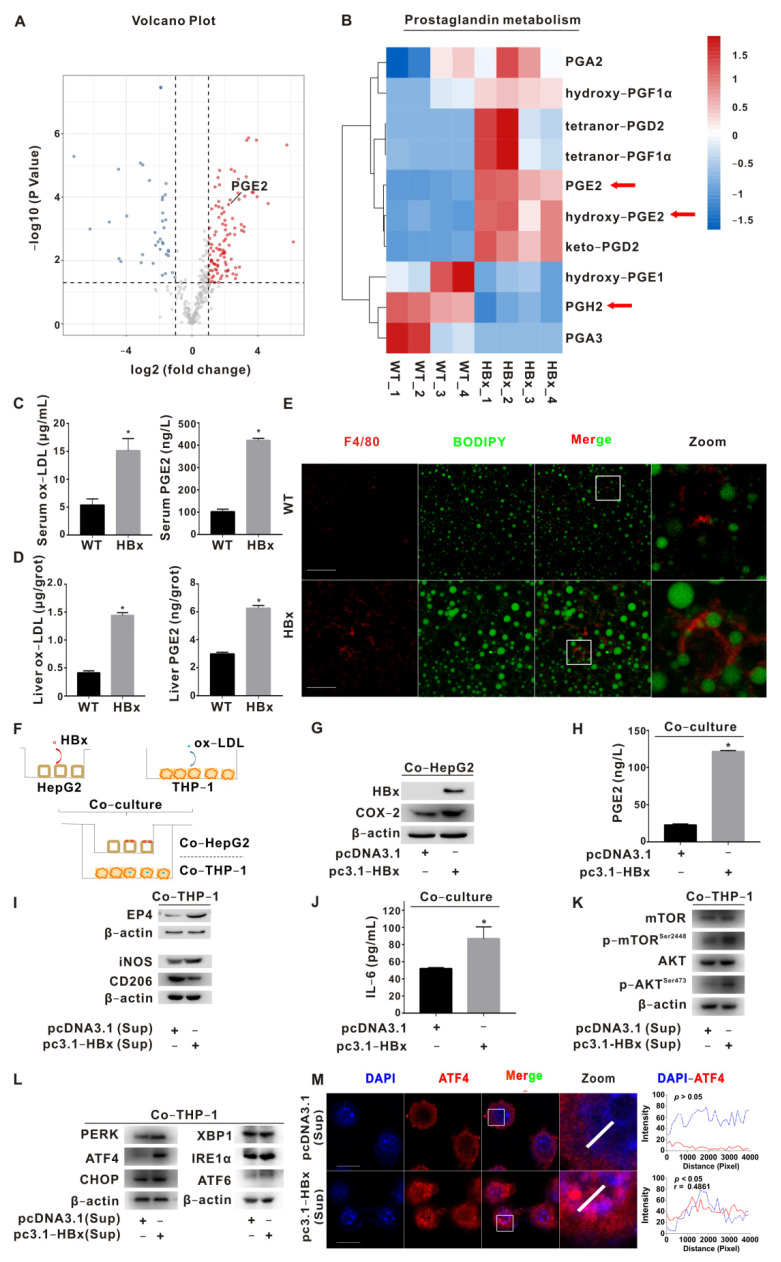
PGE2-EP4 induced ER stress-mediated macrophage polarization imbalance via mTOR signaling. (**A**,**B**) Untargeted metabolomic analysis of livers from HBx-Tg mice. (**A**) The volcano plot of differentially metabolites presents red points for upregulated metabolites (*n* = 104) and blue points for downregulated metabolites (*n* = 35). The increased level of PGE2 is shown. (**B**) The heatmap of prostaglandin metabolites in the liver tissues from HBx-Tg mice (*n* = 4) and wildtype (WT) mice (*n* = 4). Arrows indicate the PGE2-related molecules screened in this study. (**C**) ELISA detection of serum ox-LDL and PGE2 levels in HBx-Tg mice. (**D**) ELISA detection of ox-LDL and PGE2 levels in the liver tissues of HBx-Tg mice. The data are presented as the mean ± SD. * *p* < 0.05, compared with the WT group. (**E**) Representative images are shown to demonstrate the expression of F4/80 (red) and the level of lipid droplets (green). Scale bar 50 μm. (**F**–**L**) A co-culture model of HBx-expressing HepG2 cells and ox-LDL-pretreated THP-1 cells was established. (**F**) Schematic diagram of the co-culture model. (**G**) WB detection of HBx and COX-2 protein levels in co-cultured HepG2 cells. (**H**) ELISA detection of the PGE2 release level in the chamber supernatant. The data are presented as the mean ± SD. * *p* < 0.05, compared with the pcDNA3.1 group. (**I**) WB detection of EP4 and polarization marker protein levels in co-cultured THP-1 cells. (**J**) ELISA detection of the IL-6 release level in the chamber supernatant. The data are presented as the mean ± SD. * *p* < 0.05, compared with the pcDNA3.1 group. (**K**) WB detection of mTOR signaling protein levels in co-cultured THP-1 cells. (**L**) WB detection of ER stress signaling protein levels in co-cultured THP-1 cells. (**M**) Representative images are shown to demonstrate the expression of ATF4 (red) and the colocalization (purple) between ATF4 and DAPI (**Left**). DAPI (blue) was used for nucleus staining. Fluorescence profile curves were generated using Zen 2010 software (**Right**). Scale bar 10 μm.

Our previous research found that HBx combined with aflatoxin B1 triggers hepatic steatosis via COX-2 [9]. To further investigate the effect of HBx expression on hepatic metabolism, we performed metabolomic assays in the livers of HBx-Tg mice. Using untargeted metabolomics, we found that most metabolite annotations were in carbohydrate metabolism, lipid metabolism, and the digestive system (Figure S1A). Bile secretion was enriched in the KEGG pathway (Figure S1B). A total of 104 upregulated and 35 downregulated differential metabolites were found in the HBx group (Figure 1A). The levels of PGE2 and hydroxy-PGE2 in specific metabolites of prostaglandins were upregulated, and the level of the PGE2 precursor PGH2 was downregulated (Figure 1B). Since HBx-Tg mice omics suggested the activation of lipid and prostaglandin metabolism, we further tested the levels of ox-LDL and PGE2 in the serum and liver tissue. The levels of ox-LDL and PGE2 in the sera and livers of the HBx group were significantly increased (Figure 1C,D). In addition, IF experiments revealed increased levels of the macrophage marker F4/80 and lipid droplets in liver sections of the HBx group (Figure 1E).

The expression of COX-2 and mPGES-1, two key enzymes for PGE2 synthesis, were increased in the NASH liver and correlated with the NASH activity score [31]. Meanwhile, we screened the GEO database (GSE83148) and found that the mRNA level of the COX-2-encoding gene (*PTGS2*) was upregulated in the liver tissues of HBV-infected patients (Figure S1C). To explore whether PGE2 signaling is involved in macrophages in NASH, we queried the GEO database (GSE104901) to analyze the PGE2 receptor family in the liver macrophages of NASH mice induced by a high-carbohydrate, high-fat, and high-cholesterol diet. As shown in the figure, the level of *Ptger4* in the liver macrophages of NASH mice was significantly increased; in addition, biomarkers of the mTOR complex (*Mtor*), ER stress (*Atf4*, *Chop*), and polarization (*Nos2*, *Mrc1*) were also significantly different and correlated (Figure S1D,E).

Based on the clues provided by GEO, we further verified the co-culture model of HepG2 cells and THP-1 cells. A schematic diagram of the co-culture model is shown in Figure 1F. The HBx-induced upregulation of COX-2 protein levels in HepG2 cells increased the PGE2 release level and upregulation of the EP4 protein level in THP-1 cells (Figure 1G–I). In the co-cultured THP-1 cells in the HBx-expressing group, the M1 polarization marker iNOS increased, the M2 polarization marker CD206 level decreased, and the level of IL-6 in the supernatant was upregulated (Figure 1I,J). The protein levels of p-AKT^Ser473^ and p-mTOR^Ser2448^ in THP-1 cells in the HBx-expressing group increased (Figure 1K). The protein levels of ER stress markers PERK, ATF4, and ATF6 in THP-1 cells in the HBx-expressing group increased, and the level of ATF4 in the nucleus increased (Figure 1L,M). Compared with the ox-LDL+PGE2 group, the protein levels of p-AKT^Ser473^ and p-mTOR^Ser2448^ decreased after antagonizing the EP4 receptor with ONO-AE3-208 (Figure S1F); the protein levels of PERK, p-PERK^T982^, ATF4, and CHOP decreased after inhibiting mTOR with rapamycin (Rapa) (Figure S1G). After inhibiting ATF4, compared with the ox-LDL+PGE2 group, ATF4 expression and the nuclear translocalization level significantly decreased, the iNOS protein level decreased, the CD206 protein level increased (Figure S2A,B), and the level of IL-6 in the supernatant decreased (Figure S2C). The above results suggest that HBx induces COX-2-expressing hepatocyte-derived PGE2 release, activates AKT-mTOR signaling in macrophages via EP4, mediates ER stress-dependent polarization imbalance (M1), and promotes inflammatory responses.

### 2.2. NPC1 Mediated Lyso-ER Cholesterol Transport to Regulate ER Redox and ER Stress Homeostasis

**Figure 2:**
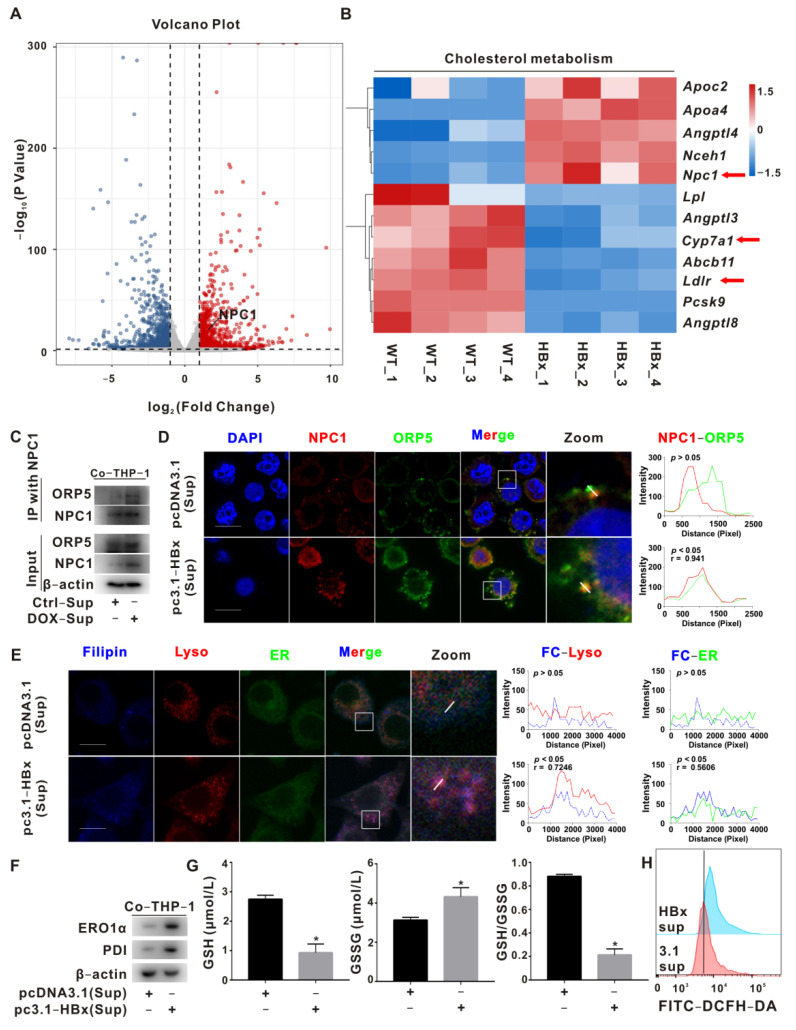
NPC1-mediated Lyso-ER cholesterol transport to regulate ER redox and ER stress homeostasis. (**A**,**B**) Transcriptomic analysis of livers from HBx-Tg mice. (**A**) The volcano plot of DEGs presents red points for upregulated genes (*n* = 859) and blue points for downregulated genes (*n* = 1066). The upregulation of the *Npc1* gene is shown. (**B**) The heatmap of DEGs (cholesterol metabolism) in the liver tissues from HBx-Tg mice (*n* = 4) and WT mice (*n* = 4). Arrows indicate the cholesterol-related genes screened in this study. (**C**) Co-immunoprecipitation detection of NPC1-ORP5 interaction level. (**D**) Representative images are shown to demonstrate the levels of NPC1 (red), ORP5 (green), and colocalization (yellow) between NPC1 and ORP5 (**Left**). Fluorescence profile curves were generated using Zen 2010 software (**Right**). DAPI (blue) was used for nucleus staining. Scale bar 10 μm. (**E**) Representative images are shown to demonstrate the levels of free cholesterol (blue), lysosome (red), ER (green), colocalization (purple) between free cholesterol and lysosome, and colocalization (cyan) between free cholesterol and ER (**Left**). Fluorescence profile curves were generated using Zen 2010 software (**Right**). Scale bar 10 μm. (**F**) WB detection of ER redox signaling protein levels in THP-1 cells. (**G**) Detection of reduced glutathione (GSH) and oxidized glutathione (GSSG) levels in THP-1 cells. The data are presented as the mean ± SD. * *p* < 0.05, compared with the pcDNA3.1 group. (**H**) Flow cytometry detection of total ROS levels in THP-1 cells.

The increased free cholesterol in the ER may induce ER redox homeostasis disturbances and mediate ER stress enhancement. To prove this point, we performed transcriptomic assays on the livers of HBx-Tg mice. There were 859 upregulated and 1066 downregulated differentially expressed genes (DEGs) in the HBx group (Figure 2A). The differentially enriched GO pathways in the HBx group included arachidonic acid metabolism, prostaglandin metabolism, cholesterol homeostasis, cholesterol metabolism, cholesterol biosynthesis, and other biological processes (Figure S3A). Hepatitis B, nonalcoholic fatty liver disease, arachidonic acid metabolism, cholesterol metabolism, and other processes were enriched in the KEGG pathway (Figure S3B). We further performed heatmap cluster analysis on cholesterol metabolism-related genes and found that the transcription level of the cholesterol transport-related gene *Npc1* was increased, and the transcription levels of the lipoprotein uptake-related receptor *Ldlr* and cholesterol hydroxylase *Cyp7a1* were downregulated (Figure 2B). In the same GEO database, the correlation analysis showed positive correlations between *Mtor*, *Npc1*, *Ero1l*, and *Atf4*, and the transcription levels of *Npc1* and *Ero1l* in the liver macrophages of NASH mice were significantly increased (Figure S3C,D).

THP-1 cells treated with ox-LDL were morphologically enlarged, and the phagosome/lysosome was significantly enlarged and increased. On this basis, the number of lipid droplets in the ox-LDL+PGE2 group was also increased, and the distance between the lysosome and ER was close (Figure S3E). Compared with the ox-LDL group and ox-LDL+PGE2 group, after Rapa inhibited mTOR, we found that NPC1 and ORP5 protein levels were decreased in the ox-LDL+PGE2 group, but not STARD3 or SLC38A9 (Figure S3F, Left); the colocalization of free cholesterol with the ER (blue-green) was decreased, and its colocalization with the lysosome (magenta) was enhanced (Figure S3G, Upper). Compared with the ox-LDL+PGE2 group, NPC1 and ORP5 protein levels decreased after si*NPC1* intervention, and fluorescence experiments revealed the diminished colocalization of free cholesterol with the ER (blue-green) and its enhanced colocalization with the lysosome (magenta) (Figure S3F, Right; Figure S3G, Lower). ERO1α, PDI, and PERK protein levels decreased after si*NPC1* intervention (Figure S3H).

Protein folding in the ER is an oxidative process that relies on protein disulfide isomerase (PDI) and ERO1α. ERO1α plays an important role in oxidative protein folding by recycling reduced PDI, so ERO1α dysfunction critically affects ER homeostasis or disease states [32]. After inhibiting ER redox with si*ERO1A*, compared with the ox-LDL+PGE2 group, the levels of ERO1α, PDI, PERK, ROS, and GSSG were decreased, and the GSH level was increased (Figure S4A–E); CD206 was increased, and iNOS and IL-6 levels in the supernatant were decreased (Figure S4A,F). Compared with THP-1-pCDH cells, ERO1α, PDI, PERK, iNOS, and IL-6 were upregulated, and CD206 was downregulated in ERO1α-overexpressing THP-1-pCDH-*ERO1A* cells (Figure S4G,H). Compared with the control group, enhanced NPC1-ORP5 interaction was detected in the IP assay, the colocalization of NPC1 and ORP5 was observed in the IF assay, and increased cytoplasmic levels of free cholesterol and enhanced ER colocalization (blue-green) were observed in the IF assay in THP-1 cells of the HBx-expressing group (Figure 2C–E); the levels of ERO1α, PDI, PERK, ROS, and GSSG were increased, and the GSH level was decreased in THP-1 cells of the HBx-expressing group (Figure 2F–H). The above results suggest that PGE2 induces mTOR signaling activation in ox-LDL-loaded macrophages, regulates NPC1-ORP5-mediated Lyso-ER cholesterol transport, and mediates M1 polarization in macrophages via ER redox and ER stress homeostasis regulation.

### 2.3. Regulation of Macrophage Polarization by mTOR-NPC1-ERO1α Pathway Was Verified in HBx-Tg Mouse Ex Vivo Assay

**Figure 3:**
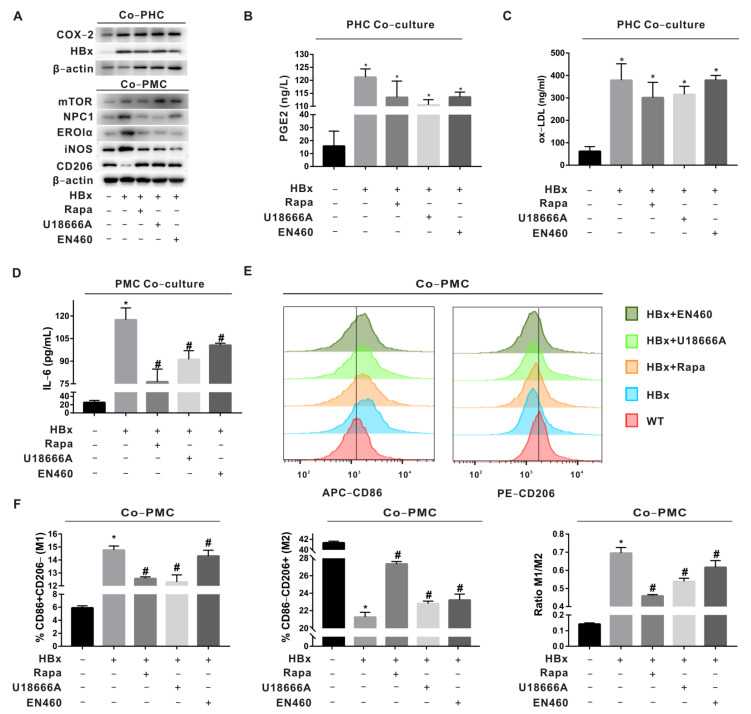
Regulation of macrophage polarization by the mTOR-NPC1-ERO1α pathway was verified in HBx-Tg mouse ex vivo assay. Primary hepatocytes (PHCs) and primary macrophages (PMCs) were co-cultured by HBx-Tg mouse liver perfusion isolation. Rapamycin (Rapa), U18666A, and EN460 were used to establish the inhibition of mTOR, NPC1, and ERO1α in PMCs. (**A**) WB detection of HBx and COX-2 protein levels in PHCs; mTOR, NPC1, ERO1α, and polarization marker protein levels in PMCs. (**B**–**D**) ELISA detection of PGE2 levels (**B**), ox-LDL levels (**C**), and IL-6 levels (**D**) in the supernatant. (**E**) Flow cytometry detection of M1/M2-polarized phenotypes. (**F**) Quantitative analysis of M1(CD86)/M2(CD206) polarization. The data are presented as the mean ± SD. * *p* < 0.05, compared with the WT group. # *p* < 0.05, compared with the HBx group.

To verify the roles of mTOR, NPC1, and ERO1α in macrophage (MC) polarization imbalance induced by HBx-expressing hepatocyte-derived PGE2, primary hepatocytes (PHCs) and primary macrophages (PMCs) were co-cultured by HBx-Tg mouse liver perfusion isolation and divided into the WT, HBx, HBx+Rapa (inhibition of mTOR in PMCs), HBx+U18666A (inhibition of NPC1 in PMCs), and HBx+EN460 (inhibition of ERO1α in PMCs) groups. In the HBx group, COX-2 was increased in PHCs; the levels of PGE2 and ox-LDL were increased in the supernatant (Figure 3A–C); iNOS was increased and CD206 was decreased in PMCs (Figure 3A); the IL-6 level was increased in the supernatant (Figure 3D); the proportion of M1 polarization (CD86+) was increased; and the proportion of M2 polarization (CD206+) was decreased (Figure 3E,F). In the groups with mTOR, NPC1, and ERO1α interventions, the above indicators were rescued to some extent, suggesting that PGE2/ox-LDL in HBx-expressing mouse livers mediates intercellular communication between PHCs and PMCs, regulates mTOR-NPC1 in PMCs to regulate Lyso-ER cholesterol transport, and induces ER stress-dependent PMC polarization imbalance (M1).

### 2.4. EP4 Intervention Reduced the HBx-Related Lipotoxicity and NASH In Vivo

**Figure 4:**
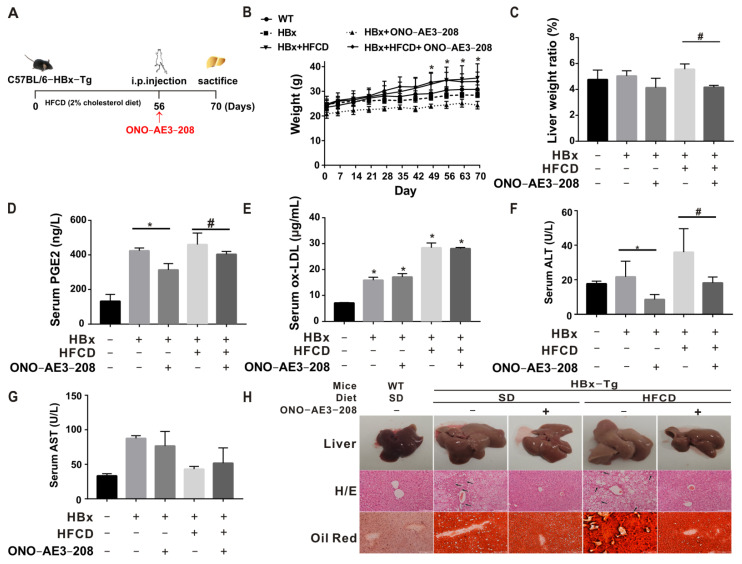
EP4 intervention reduced HBx-related lipotoxicity and NASH in vivo. C57BL/6-HBx-Tg (transgenic) mice and wildtype (WT) mice were used to establish related models fed a high-fat/cholesterol diet (HFCD) or the standard diet and administered ONO-AE3-208 (5 mg/kg, intraperitoneal injection, once every other day for 2 weeks) for EP4 intervention. (**A**) Experimental animal model and treatment model diagram in mice (*n* = 3 in each group). (**B**) Body-weight growth curve. (**C**) Liver/body-weight ratio. (**D**,**E**) ELISA detection of PGE2 level (**D**) and ox-LDL level (**E**) in serum. (**F**,**G**) Detection of ALT and AST in serum. (**H**) Representative images of the NAFLD/NASH phenotypes (liver morphology, H/E staining, and Oil Red O staining). Scale bar 100 μm. Black arrows indicate inflammatory foci. The data are presented as the mean ± SD. * *p* < 0.05, compared with the WT or HBx group. # *p* < 0.05, compared with the HBx + HFCD group.

The experimental model and treatment pattern of mice for in vivo tests are shown in Figure 4A. In the model construction, there was a tendency toward inhibited body-weight gain in the HBx group, with significant weight gain after 49 days of feeding a high-fat/cholesterol diet (HFCD), suggesting an abnormal metabolic pattern in HBx-Tg mice (Figure 4B). Although there was no significant change in the overall liver/body-weight ratio, there was an increasing trend in the HBx + HFCD group and a significant decrease after intervention with ONO-AE3-208, suggesting that EP4 intervention could rescue HBx + HFCD-induced liver injury (Figure 4C). Compared with the WT group and the corresponding control groups, PGE2, ox-LDL, and ALT in the serum were elevated in the HBx and HBx + HFCD groups and decreased in the HBx + ONO and HBx + HFCD + ONO groups (Figure 4D–G). A further test of relevant indicators in liver homogenates showed that COX-2 protein expression levels were upregulated (Figure S5A), while PGE2, ox-LDL, IL-6, TG, TC, and FC levels in the liver were elevated in the HBx and HBx + HFCD groups and decreased in the HBx + ONO and HBx + HFCD + ONO groups (Figure S5B–E). The HBx and HBx + HFCD groups showed the ballooning of hepatocytes, vacuolization, and inflammatory foci, increased Oil Red O–stained lipid droplets, and increased NASH scores; the ONO-AE3-208 intervention group showed the reduced liver pathological characteristics and the decreased NASH scores (Figure 4H, Table 1). These results suggest that EP4 intervention can reduce HBx-dependent lipid accumulation and inflammation-related liver injury in NASH phenotypes in mice.

### 2.5. EP4 Intervention Rescued Hepatic Macrophage Polarization Imbalance In Vivo

**Figure 5:**
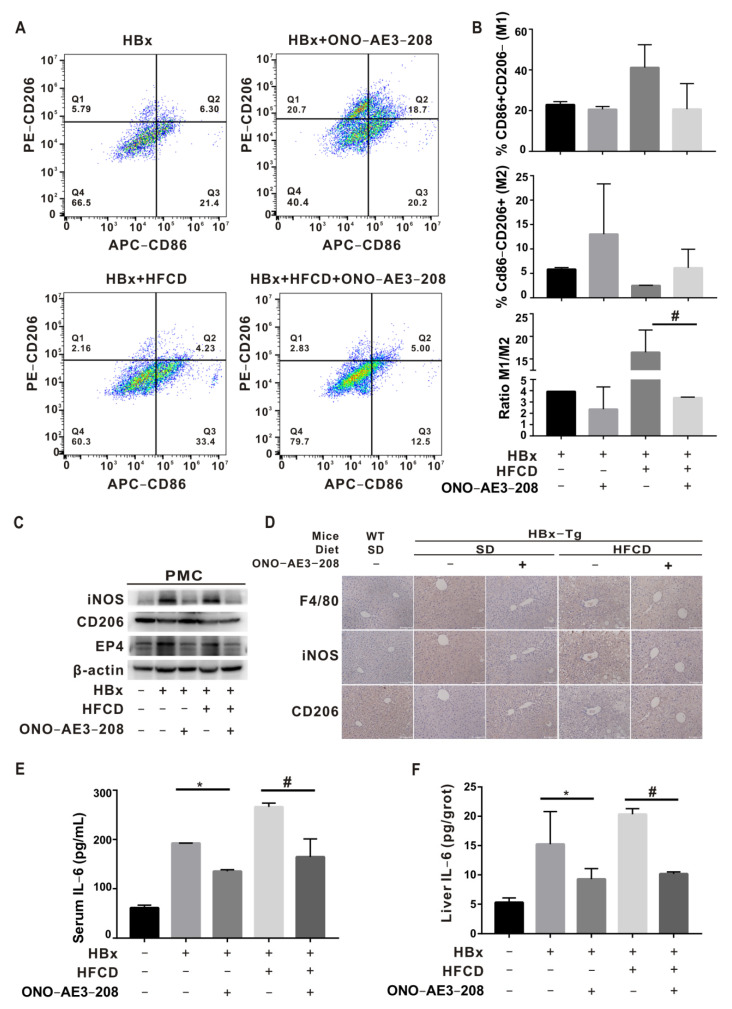
EP4 intervention rescued hepatic macrophage polarization imbalance in vivo. (**A**–**C**) PMCs were isolated by liver perfusion from different HBx-Tg mouse models. (**A**) Flow cytometry of the M1/M2 polarization phenotype in mononuclear-derived Kupffer cells. (**B**) Quantitative analysis of M1(CD86)/M2(CD206) polarization. (**C**) WB detection of EP4, iNOS, and CD206 in PMCs. (**D**) Representative images of the IHC staining of F4/80, iNOS, and CD206 in liver tissue sections are shown. Scale bar 100 μm. (**E**,**F**) ELISA detection of IL-6 levels in serum (**E**) and livers (**F**). The data are presented as the mean ± SD. * *p* < 0.05, compared with the HBx group. # *p* < 0.05, compared with the HBx + HFCD group.

**Figure 6:**
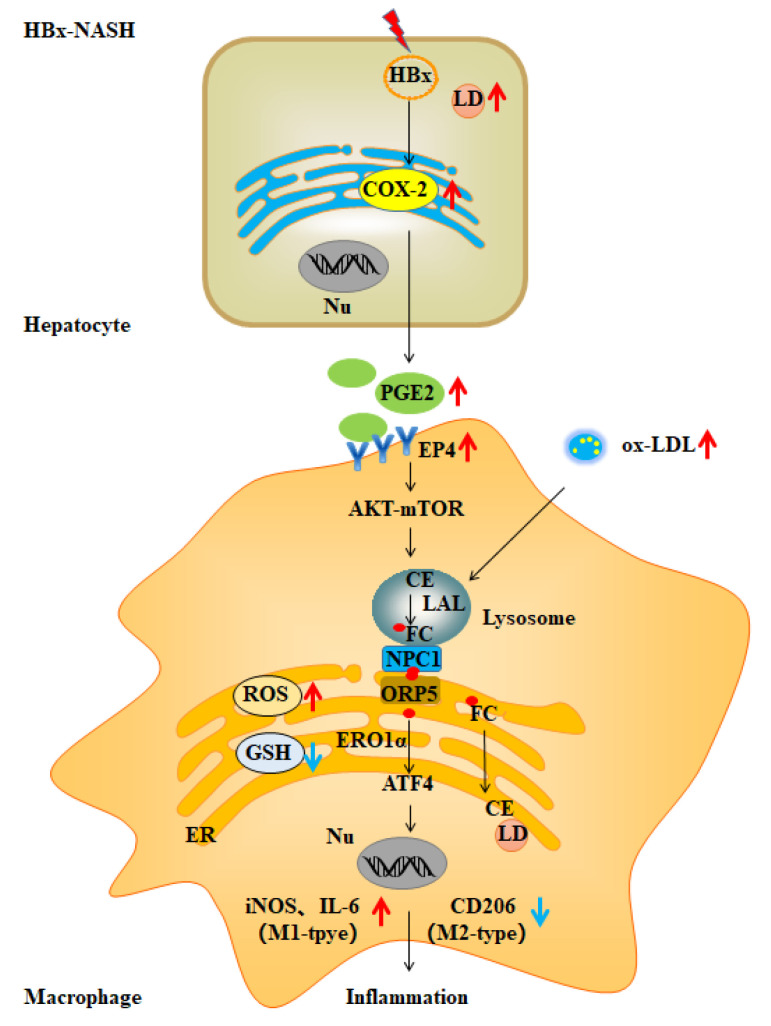
The mechanism of Lyso-ER cholesterol-transport-regulated macrophage polarization in HBx-related NASH. In HBx-induced ox-LDL-dependent NASH-associated inflammatory responses, the PGE2-EP4-mTOR signaling axis regulated NPC1-ORP5 interactions mediating Lyso-ER cholesterol transport. ER-redox-dependent ER stress induced by ER cholesterol is involved in mediating the M1 polarization of macrophages. The targeted intervention of EP4 rescues MC polarization imbalance to improve the NASH phenotype in HBx-Tg mice. The red arrows indicate the index was increased and the blue arrows indicate the index was decreased in this study.

PMCs were further isolated by liver perfusion from different HBx-Tg mouse models to investigate the regional immunoregulatory function of liver macrophages. In the HBx-Tg and HBx-Tg + HFCD groups, the levels of p-AKT^ser473^, p-mTOR^ser2448^, NPC1, ORP5, ERO1α, PDI, PERK, and ATF4 proteins increased in PMCs (Figure S6A,B,E); NPC1 and ORP5 protein levels increased in the liver (Figure S6C); cytoplasmic levels of free cholesterol and enhanced ER colocalization (blue-green) increased in PMCs (Figure S6D); the proportion of F4/80^+^/CD11b^+^ mononuclear-derived KC increased (Figure S6F); the proportion of M1 polarization of CD86^+^/CD206^−^ increased, and the proportion of M2 polarization of CD206^+^/CD86^−^ decreased (Figure 5A,B); EP4 and iNOS protein expression levels increased, and CD206 protein expression levels decreased in PMCs (Figure 5C); F4/80 and iNOS increased, and CD206 decreased in the liver (Figure 5D); and IL-6 was elevated in the serum and liver (Figure 5E,F). Moreover, after the ONO-AE3-208-induced inhibition of EP4, there was some improvement in the above indicators. Altogether, HBx-Tg mice subjected to HFCD exposure expressed HBx-associated NASH phenotypes; in the in vivo assay, it was demonstrated that HBx-expressing hepatocyte-derived PGE2 mediated hepatic MC polarization dysregulation via the mTOR-NPC1-ER stress signaling pathway; and EP4 receptor-targeted intervention rescued HBx-associated NASH mediated by hepatic MC polarization imbalance (Figure 6).

## 3. Discussion

In the present study, we found an improved MC polarization imbalance after the inhibition of ERO1α and ATF4 in cholesterol-loaded macrophages, suggesting that ER redox and ER stress may mediate hepatic immune inflammatory responses by regulating MC polarization. Transient ER stress, a defense mechanism, protects hepatocytes from overexposure to nutrients such as lipids and/or exogenous agents such as viruses. Moreover, the chronic activation of ER stress is a central process in the transition from the simple steatosis stage to the NASH stage in NAFLD, including triggering cell death, inducing inflammatory responses, and accelerating metabolic disorders [33]. The initiation-sensing proteins of ER stress, PERK, IRE1α, and ATF6 inhibitors can alleviate the activation of cell death and inflammatory pathways as well as metabolic disorders. So, ER stress may be a new idea and a promising target for NASH intervention and treatment [34]. The latest study indicated that ER stress in innate immune cells contributes to NASH. For example, ATF6, by mediating a proinflammatory synergy between ER stress and TLR activation, is involved in the development of liver injury [35]. XBP1-mediated ER stress promoted steatohepatitis via NLRP3 inflammatory vesicle-mediated M1-type polarization [6]. Therefore, the interaction between liver inflammation and ER stress and the elucidation of ER stress-related mechanisms in macrophages can contribute to the prevention and treatment of NASH.

In this study, we found that the inhibition of cholesterol transport from lysosomes to ER alleviated ER cholesterol accumulation-induced ER redox and ER stress by interfering with NPC1-ORP5, suggesting that NPC1-ORP5-mediated cholesterol transport between Lyso and ER may regulate hepatic immunometabolism. The downregulation of NPC1-ORP5 and the inhibition of cholesterol transport from Lyso to the ER were found by interfering with mTOR, suggesting that NPC1-ORP5-mediated cholesterol transport between Lyso and the ER may be regulated by mTOR. The role of Lyso-ER cholesterol transport-related molecules in macrophage polarization has been poorly reported and is an area that remains to be elucidated. Xu et al. found that simvastatin upregulates NPC1 via CYP7A1/LXRα signaling in ox-LDL-loaded macrophages to promote FC efflux from the lysosome, reduce the secretion of proinflammatory cytokines, and inhibit the M1 polarization phenotype, which is important for atherosclerosis intervention [36]. Borthwick et al. found that the overexpression of STARD3 caused significant upregulation of the receptor ABCA1 in macrophages, alleviated cholesterol ester accumulation by promoting cholesterol efflux, and suggested that enhanced lysosomal cholesterol transport induces an anti-atherogenic macrophage lipid phenotype [37]. The overexpression of ORP1L in macrophages causes decreased ABCG1 expression, and impaired cholesterol efflux promotes inflammatory signaling, leading to an M1-type polarization shift and exacerbating atherosclerotic lesions in *Ldlr*^−/−^ mice [38]. In addition, it has been suggested that ox-LDL-induced lysosomal cholesterol accumulation plays a specific role in triggering inflammation and is a driver of atherosclerosis and NASH, and further attention should be paid to how to stimulate lysosomal cholesterol transport and block ox-LDL particle uptake interventions to prevent cholesterol accumulation in the lysosome [39]. Head et al. found that NPC1 mediates cholesterol transport via mTORC1 to promote angiogenesis [40]. These studies suggest that Lyso-ER cholesterol transport regulates the proinflammatory phenotype of foam macrophages, which is also associated with mTORC1.

In this study, we found that the HBx expression of hepatocyte-derived PGE2 induced the upregulation of M1-type polarization and the downregulation of M2-type polarization in macrophages via EP4 receptors, suggesting that PGE2/EP4 may mediate communication between hepatic HC and MC cells to regulate the local immune microenvironment in the liver. PGE2 has been shown to influence NAFLD progression in insulin resistance, hyperglycemia, hepatic lipid accumulation, and inflammation [41]. The regulatory effect of PGE2 on macrophage polarization is not invariable and may be related to the specific microenvironment [42,43]. The EP4 receptor has been well described as an effective target for possible interventions in prostate, liver, colon, breast, skin, and vulvar cancers; NSAID-induced enteropathy; bone resorption, metabolism, and formation; atherosclerotic inflammation; rheumatoid arthritis; and other diseases [44]. The EP4 antagonist ONO-AE3-208 has been shown to inhibit malignant tumor invasion, migration, and metastasis [45]. However, as of today, no studies related to EP4 as an intervention target for NAFLD or NASH have been reported. Our previous study confirmed that COX-2 expression was upregulated in aflatoxin B1-treated hepatocytes and induced inflammatory liver injury. The inhibition of COX-2 with celecoxib improved the local immune microenvironment of the liver and blocked inflammatory injury [22]. On this basis, we proceeded to find that ONO-AE3-208, as an EP4 receptor antagonist of PGE2 inhibition, could improve the disturbance of hepatic lipid accumulation and KC polarization balance in mice to some extent, attenuate inflammatory injury, and ultimately rescue the HBx-associated NASH phenotype, suggesting for the first time that the EP4 receptor of PGE2 may become a potential target for intervention.

In this study, we found that by improving the disturbance of hepatic cholesterol accumulation and KC polarization balance, we were able to reduce inflammatory injury and ultimately rescue the HBx-associated NASH phenotype, suggesting that the dysregulation of cholesterol homeostasis and MC polarization can be used as biomarkers of HBx-associated NASH immune injury. Reduced high-density lipoprotein cholesterol (HDL-C) and an elevated monocyte-to-HDL-C ratio (MHR) are significantly associated with mortality in HBV patients and can be used as prognostic biomarkers [46,47]. There is a complex association between the polarized phenotype of macrophages and HBV infection, with M1 activation indicating a strong immune response to HBV infection and M2 indicating persistent HBV infection, which is associated with disease progression [48].

In summary, we identified that PGE2-EP4-mTOR signaling regulates NPC1-ORP5 interaction mediating Lyso-ER cholesterol transport. ER-redox-dependent ER stress regulation induced by ER cholesterol is involved in mediating the M1 polarization of macrophages in HBx-induced ox-LDL-dependent NASH-associated inflammatory responses.

## 4. Materials and Methods

### 4.1. Bioinformatics Analysis

HBx-Tg mice were constructed as mentioned in our previous study [49]. For the transcriptome and untargeted metabolomic analyses of mouse liver, the complete liver lobes of mice in the WT group and the HBx group were used, with 2 mice in each group, and each liver lobe was tested twice, with a total of 8 samples. For processing and testing, the samples were entrusted to NovoGen Medical (Beijing, China). After the samples for transcriptomics were pretreated, mRNA extraction and detection, library construction and quality control, and up-sequencing were performed to obtain mRNA sequence information. Statistical methods such as DESeq, the negative binomial distribution model, and the padj BH value were used to compare gene mRNA-level differences and screen-related genes. The analysis process included differential significance analysis, GO function enrichment analysis, and KEGG pathway enrichment analysis. Using non-targeted metabolomics based on high-resolution mass spectrometry detection technology, we detected the characteristic peaks of the sample molecules and matched the metabolite identified in biological systems with the mzCloud database constructed by standards and with MassList and mzVault databases. The specific process included simple screening by the retention time, mass-to-charge ratio, and other parameters, adjusting the peak alignment according to the retention time and mass deviation, and then extracting the peak based on the information of the given mass deviation, signal-to-noise ratio, and additive ions. Then, the identified metabolites were compared with the database. Metabolites with a coefficient of variation of less than 30% in the quality control samples were retained as the final identification results. Finally, the identified metabolites were annotated with functions and classifications using databases such as KEGG. Multivariate statistical methods, such as principal component analysis and partial least-squares discriminant analysis, were used to filter the differential metabolites by downscaling and regression analysis. The raw histological data were normalized and, using the online bioinformatics website (https://hiplot.com.cn/, accessed on 2 April 2022), visualized as volcano maps, heat maps, and bubble maps.

Comprehensive gene expression dataset analysis included the analysis of the transcript levels of the COX-2 gene (*PTGS2*) involved in the catalytic synthesis of PGE2 from liver tissues of HBV-infected patients in the GEO database (GSE83148) and the analysis of the transcription of PGE2 receptors, mTOR components, lysosomal cholesterol transporters, ER redox and stress, and M1/2 polarization markers in NASH mouse liver macrophages in the GEO database (GSE104901). Differential genes were screened out to provide clues for subsequent experiments.

### 4.2. Reagents and Antibodies

PGE2 was purchased from Santa Cruz Biotechnology (Santa Cruz, CA, USA); ox-LDL was purchased from Guangzhou Yiyuan Bio (Guangzhou, China); U18666A and EN460 were purchased from MedChemExpress (Monmouth Junction, NJ, USA); ONO-AE3-208 was purchased from CSNpharm (Arlington Heights, IL, USA); si*NPC1*, si*ERO1A*, si*ATF4*, and siNC were purchased from RiboBio (Guangzhou, China); Rapa was purchased from Sigma (St. Louis, MO, USA); and doxycycline hydrochloride was purchased from Macklin (Shanghai, China).

Primary antibodies: Anti-ERO1α, -EP4, -iNOS, -CD206, and -XBP1 were purchased from Abcam (Waltham, MA, USA); anti-PERK, -mTOR, -p-mTOR^Ser2448^, and -IRE1α were purchased from Cell Signalling Technology (Danvers, MA, USA); anti-NPC1, -STARD3, -HBx, and -β-actin were purchased from Santa Cruz Biotechnology (Santa Cruz, CA, USA); anti-ORP5 was purchased from Bioss (Beijing, China); anti-SLC38A9 was purchased from NOVOS Biologicals (Littleton, CO, USA); anti-COX2 was purchased from HUABIO (Hangzhou, China); anti-Akt and -p-AKT^Ser473^ were purchased from Affinity Biosciences (Liyang, China); anti-ATF4 was purchased from Proteintech (Chicago, IL, USA); anti-CHOP was purchased from Ruiying Biological (Suzhou, China); and anti-ATF6 and -p-PERK^T982^ were purchased from ABclonal Technology (Wuhan, China). Secondary antibodies: Anti-mouse IgG (H+L) was purchased from Invitrogen (Carlsbad, CA, USA), anti-rabbit IgG (H+L) was purchased from Thermo Scientific (Waltham, MA, USA), and fluorescent secondary antibodies 488-labeled and 647-labeled IgG were purchased from Beyotime (Shanghai, China).

### 4.3. Cell Grouping and Processing

HBx-expressing hepatocytes: With pcDNA3.1 plasmid as a control, HepG2 cells were transfected with a pcDNA3.1-HBx plasmid (1 μg) for 8 h and then recovered for 1 day; with dimethyl sulfoxide as a control, the expression of HBx was induced in HepG2-Teton-HBx cells by treatment with doxycycline hydrochloride (1 μg/mL, 2d).

Co-culture of hepatocytes and macrophages: HepG2 cells recovered from transfection were plated on the upper layer of the chamber, and THP-1 cells pretreated with ox-LDL (1 μg/mL, 1d) were plated on the lower layer of the chamber.

Culturing and processing of mononuclear-derived macrophage THP-1: THP-1 cells were treated with phorbol ester (100 ng/mL, 1 d) to induce their differentiation into macrophages. Using dimethyl sulfoxide as a control, cells were treated with ox-LDL (50 μg/mL) for 1 day to build a cholesterol-load model, ox-LDL+PGE2 (0.25 μM) for 1 day to establish a liver injury exposure model, ox-LDL+PGE2+ONO-AE3-208 (1 μM) for 1 day to construct the EP4 intervention model, and ox-LDL+PGE2+Rapa (50 nM) for 1 h to construct the mTOR intervention model. With siNC as a control, cells were transfected with ox-LDL+PGE2+si*NPC1* (50 nM) for 8 h and recovered for 1 day to construct the NPC1 intervention model, ox-LDL+PGE2+si*ERO1A* was used to construct the ERO1α intervention model, and ox-LDL+PGE2+si*ATF4* was used to construct the ATF4 intervention model. The pcDH-*ERO1A* cell line with high expression of ERO1α was established with the pcDH vector as the control.

### 4.4. Western Blotting (WB)

Western blotting analysis was performed as previously described [50]. According to the standard procedures commonly used in the laboratory, detection operations were performed: gel preparation, electrophoresis, transfer, blocking, primary antibody incubation, secondary antibody incubation, and luminescence development.

### 4.5. Enzyme-Linked Immunosorbent Assay (ELISA)

According to the corresponding experimental design, when approaching the end point of the treatment, referring to the instructions of the kit, PGE2 (MEIMIAN, Yancheng, China), IL-6 (SINOBESTBIO, Shanghai, China), and ox-LDL (RUIDA HENG HUI, Beijing, China) were measured by a multi-function microplate reader at 450 nm.

### 4.6. Immunofluorescence (IF)

Immunofluorescence analysis was performed as described previously [51]. Cells were incubated with the lipid droplet probe (BODIPY™ 493/503, green) (Thermo Scientific, MA, USA), free cholesterol probes (Filipin complex, blue) (Sigma, MO, USA), lysosome probes (Lyso-Tracker, red) (Molecular Probes, Waltham, MA, USA), endoplasmic reticulum probes (ER-Tracker, green) (Molecular Probes, MA, USA), or target protein antibody or nuclear dye DAPI near the end of the treatment according to the appropriate experimental design. A high-sensitivity laser confocal microscope (LSM 880, Zeiss, Jena, Germany) was used to observe and analyze the colocalization of free cholesterol and the lysosome or ER, the expression and colocalization of NPC1 and ORP5, and the level of ATF4 in the nucleus.

### 4.7. Flow Cytometry (FCM)

According to the corresponding experimental design, the DCFH-DA probe (Beyotime, Shanghai, China) was incubated for 30 min according to the kit instructions at the end of the treatment, and the green fluorescence intensity was recorded to detect the ROS level by the FITC channel parameter using a flow cytometer.

### 4.8. Transmission Electron Microscopy (TEM)

Transmission electron microscopy analysis was performed as described previously [51]. Referring to standard procedures commonly used in the laboratory, electron microscopy samples were prepared near the end of the treatment process and sent to the electron microscopy room to observe the morphology of lysosomes and the ER.

### 4.9. Total Glutathione–Oxidized Glutathione Test Kit

According to the corresponding experimental design, the operation was carried out according to the kit (NJJCBIO, Nanjing, China) instructions at the end of the treatment. Reduced glutathione (GSH) = Total glutathione − 2 × oxidized glutathione (GSSG).

### 4.10. Isolation of Hepatic Macrophages by Mouse Hepatic Perfusion

Basic operations were performed as described previously [22]. In brief, the mice were anesthetized by intraperitoneal injection of 10% urethane with 1% body weight. The calcium-free perfusate was slowly injected until the liver was swollen. The collagenase perfusate was slowly injected. The perfusion was stopped and followed by digestion for 10 min at 37°C. After filtration with a cell strainer and centrifugation at 70× *g* for 3 min, the precipitate was primary hepatocytes (PHCs), and the supernatant (non-parenchymal cells) was taken to further isolate primary macrophages (PMCs). The supernatant was centrifuged at 500× *g* for 3 min, the supernatant was removed, and the pellet was resuspended; this process was repeated twice. The pellet was resuspended in 5 mL of the medium, the suspension was centrifuged at 50× *g* for 3 min, and the supernatant was transferred; this process was repeated twice. Supernatants were collected and centrifuged at 500× *g* for 3 min for a total of 3 times, and the precipitate was PMCs.

### 4.11. Co-Culture of Mouse PHCs and PMCs

The PHCs of the WT group and the HBx group were placed on the upper layer of the chamber, and PMCs were placed on the lower layer of the chamber. After adhering to the wall, Rapa or U18666A and EN460 were added as a treatment for 1 day.

### 4.12. Animal Model and Diets

Eight–twelve-week-old C57BL/6 and C57BL/6-HBx-Tg mice were randomly divided into 5 groups (*n* = 5): the control group (wild type), HBx group, HBx + HFCD group (high-fat and high-cholesterol diet for 10 weeks, positive NAFLD/NASH model group), HBx + ONO-AE3-208 group (5 mg/kg, intraperitoneal injection, once every other day for 2 weeks, EP4 intervention group), and HBx-Tg + HFCD + ONO-AE3-208 group. The animals were kept in an independent ventilation cage system to ensure sufficient water and food and a clean environment. The whole process complied with the normative requirements of the Xiamen University Laboratory Animal Management Ethics Committee.

### 4.13. Serum Index Quantification

Referring to the kit instructions, the levels of ALT (NJJCBIO, Nanjing, China), AST (NJJCBIO, Nanjing, China), ox-LDL, PGE2, and IL-6 were detected.

### 4.14. Liver Homogenate Index Quantification

Referring to the kit instructions, the levels of ox-LDL, TG, TC, FC (APPLYGEN, Beijing, China), PGE2, and IL-6 were detected and corrected with the protein concentration.

### 4.15. FCM Detection and Analysis of the Polarization Ratio of Mononuclear-Derived KC

Block Fc receptors: A total of 0.5–1 μg of CD16/32 monoclonal antibody was added and incubated at room temperature for 15 min. Cell staining: A total of 5 μL each of flow-through antibodies PerCP/Cyanine5.5-F4/80, FITC-CD11b, PE-CD206, and APC-CD86 (Elabscience, Wuhan, China) was added, mixed, and incubated for 45 min in the dark. The cells were resuspended in PBS with 1% BSA, the cell suspension was centrifuged at 300× *g* for 5 min, and the supernatant was discarded. Cells were resuspended in 1% BSA in PBS and filtered into flow tubes for detection and analysis by flow cytometry.

### 4.16. Histopathological Observation of Liver

Liver samples of suitable size were fixed in 4% paraformaldehyde at 4°C overnight; then, the paraformaldehyde was replaced, and the samples were used to prepare paraffin sections. Liver samples of suitable size were embedded in OCT gel, placed at −80°C overnight, and then used to prepare frozen sections.

Immunofluorescence (IF) assay: Frozen sections were taken, incubated with a lipid droplet probe (BODIPY™ 493/503, Thermo Scientific, MA, USA) and antibody to the macrophage marker F4/80, and the expression and distribution of lipid droplet levels were observed and analyzed with F4/80 using a high-sensitivity laser confocal microscope (LSM 880).

Immunohistochemistry (IHC) assay: IHC experiments were performed using IHC kits (Maxim, Fuzhou, China), and the expression of related proteins was observed by incubating cells with anti-F4/80, -iNOS, -CD206, -NPC1, and -ORP5 according to standard laboratory procedures.

Hematoxylin–eosin staining: Paraffin sections were taken following the standard laboratory procedures for dewaxing and hydration, hematoxylin staining, eosin staining, dehydration, and mounting to observe the level of liver tissue damage by microscopic examination.

Oil Red O staining: Frozen sections were taken, and the level of lipid accumulation in the liver was observed according to the instructions of the kit (NJJCBIO, Nanjing, China).

NASH scoring [52]: According to the analysis of histological features in the slices, the degree of liver steatosis was divided into 4 grades (0–3), the number of inflammatory reaction foci was divided into 4 grades (0–3), and the degree of hepatocyte damage (balloon degeneration) was divided into 3 grades (0–2). A total score of 0–3 was judged as non-NASH (N), while a score of 4–8 was considered NASH.

### 4.17. Statistical Analysis

GraphPad Prism 7.0 and CorelDRAW-X4-SP2 software were used to analyze and draw charts, in which the data are presented in the form of mean ± standard deviation (SD). The specific statistical analysis methods were as follows: An independent sample *t*-test was used for the comparison of two groups of data. One-way analysis of variance was used for data comparison between multiple groups. Further analysis of the differences between pairs was performed by Dunnett’s *t*-test. The correlation was analyzed by Pearson’s correlation coefficient, and *p* < 0.05 means that the difference is statistically significant.

## Figures and Tables

**Table 1:** NASH scoring for the HBx-related NASH and EP4 intervention in vivo.

Mice	WT	HBx-Tg			
Diet	SD	SD		HFCD	
ONO-AE3-208	−	−	+	−	+
Steatosis	0	2	1	3	1
Lobular inflammation	0	2	0	3	0
Hepatocyte ballooning	0	2	0	2	1
NAFLD activity score	0	6	1	8	2
Diagnostic classification	N	NASH	N	NASH	N

Note: SD, standard diet; N, non-NASH.

## Data Availability

The GEO omics database (GSE1049001 and GSE83148) can be consulted and downloaded from https://www.ncbi.nlm.nih.gov/gds/, accessed on 2 April 2022. The rest of the relevant data are contained within this article and in the supporting information.

## Supplementary Materials

The following supporting information can be downloaded at: https://www.mdpi.com/article/10.3390/ijms231911660/s1.

## Abbreviations

COX-2, cyclooxygenase 2; ERO1α, endoplasmic reticulum oxidoreductase-1-like protein α; EP4, PGE2 receptor 4; ER, endoplasmic reticulum; FC, free cholesterol; FCM, flow cytometry; HBx, hepatitis B virus x protein; HBV, hepatitis B virus; HDL-C, high-density lipoprotein cholesterol; HFCD, high-fat/cholesterol diet; IF, immunofluorescence; IHC, immunohistochemistry; LAL, lysosomal acid lipase; LDL, low-density lipoprotein; Lyso, lysosome; mTOR, mammalian target of rapamycin; MAM, mitochondria-associated ER membrane; MCS, membrane contact sites; MHR, monocyte-to-HDL-C ratio; NAFLD, nonalcoholic fatty liver disease; NASH, nonalcoholic steatohepatitis; NPC1, Niemann–Pick type C1; ORP5, oxysterol-binding protein-related protein 5; ox-LDL, oxidized low-density lipoprotein; PDI, protein disulfide isomerase; PGE2, prostaglandin E2; PHC, primary hepatocyte; PMC, primary macrophage; Rapa, rapamycin; SD, standard deviation; START, steroidogenic acute regulatory protein-related lipid transfer domain; TEM, transmission electron microscopy; WB, Western blotting.
