# Supplementary material for: Hepatocyte-Derived Prostaglandin E2-Modulated Macrophage M1-Type Polarization via mTOR-NPC1 Axis-Regulated Cholesterol Transport from Lysosomes to the Endoplasmic Reticulum in Hepatitis B Virus x Protein-Related Nonalcoholic Steatohepatitis
Source: Int J Mol Sci. 2022 Oct 1;23(19):11660. doi: 10.3390/ijms231911660 (PMC9569602; doi:10.3390/ijms231911660)
Supplement: Supplementary file 1 [file ijms-23-11660-s001.zip › ijms-1920711-supplementary.pdf]

Hepatocyte-Derived Prostaglandin E2-Modulated Macrophage M1-Type Polarization via mTOR-NPC1 Axis-Regulated Cholesterol Transport from Lysosomes to the Endoplasmic Reticulum in Hepatitis B Virus x Protein-Related Nonalcoholic Steatohepatitis

You Lan [#], Bo Qian [#], Hai-Yan Huang, Pan Wang, Ting Li, Qi Yuan, Han-Yu Zhang, Yu-Chun Lin [*] and Zhong-Ning Lin [*]

[1] State Key Laboratory of Molecular Vaccinology and Molecular Diagnostics, School of Public Health, Xiamen University, Xiamen, 361102, China
[#] These authors contributed equally to this work.
* Correspondence: linych@xmu.edu.cn (Y.-C.L.) and linzhn@xmu.edu.cn (Z.-N.L.); Tel: +86-592-2880615 (Z.-N.L.)

Supplementary Materials:

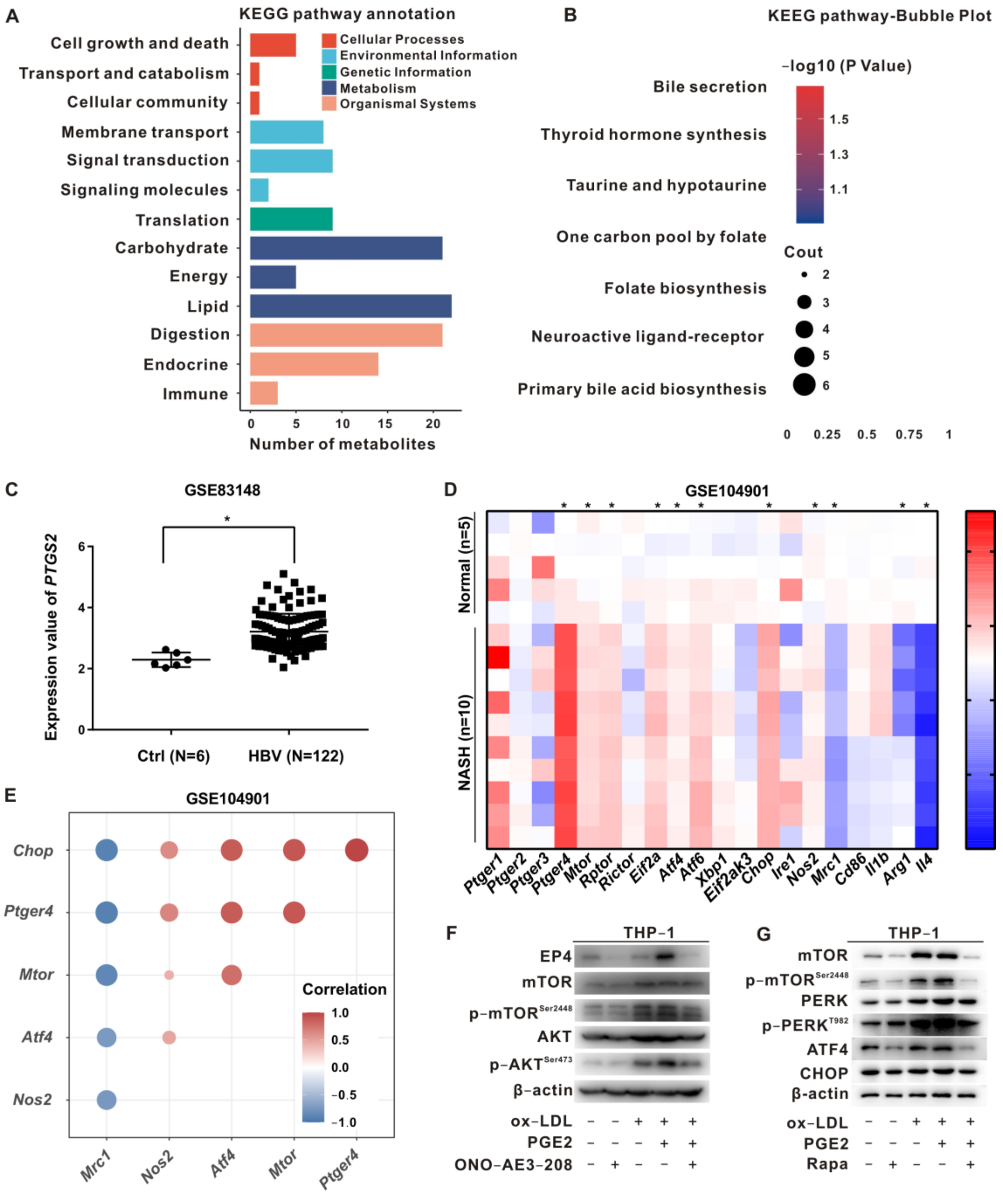

Figure S1. PGE2-EP4 induced ER stress-mediated macrophage polarization imbalance via mTOR signaling. (A, B) Untargeted metabolomics analysis of HBx-Tg mice liver. (A) KEGG pathway annotation for analysis of metabolites. (B) KEGG pathway enrichment was displayed for analysis of differential metabolites. (C) The GEO database (accession no. GSE83148) analyzes *PTGS2* expression levels of liver tissues from HBV-infected patients (n = 122). * $p < 0.05$, compared with the Ctrl group (n = 6). (D, E) GEO database (accession no. GSE104901) analyzes gene expression levels in liver macrophages from the NASH mice. (D) The heatmap of the PGE2 receptor family, mTOR complex, ER stress, and polarization marker genes in liver macrophages from the NASH mice (n = 10) and the normal mice (n = 5). * $p < 0.05$, compared with the normal group. (E) Pearson's correlation analysis for the relationship between mRNA levels of *Ptger4*, *Mtor*, *Atf4*, *Chop*, *Nos2*, and *Mrc1*. (F) The targeted intervention effect of ONO-AE3-208 on PGE2-induced mTOR activation in ox-LDL loaded THP-1 cells. Expression levels of EP4, AKT-mTOR were detected by WB. (G) The targeted intervention effect of Rapa on PGE2-induced ER stress in ox-LDL loaded THP-1 cells. Expression levels of mTOR and ER stress signals were detected by WB.

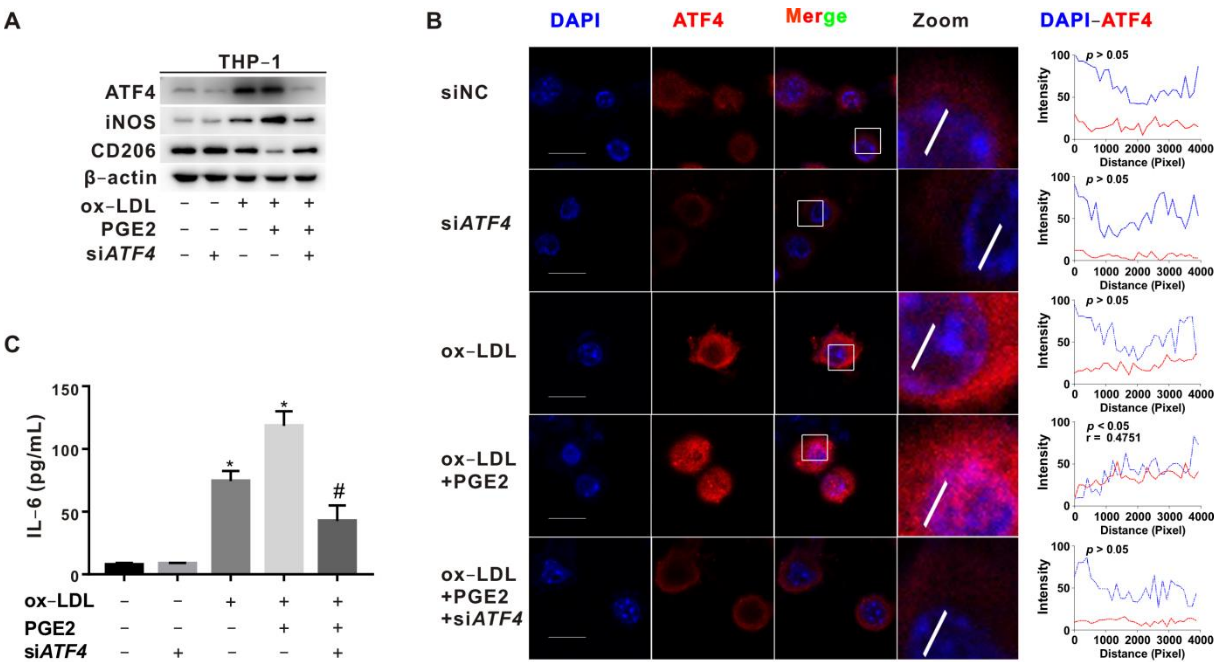

Figure S2. The targeted intervention effect of si*ATF4* on PGE2-induced polarization imbalance in ox-LDL loaded THP-1 cells. (A) WB detection of ATF4 and polarization marker protein levels. (B) Representative images for evaluating the expression of ATF4 (red) and the colocalization (purple) between ATF4 and DAPI (Left). DAPI (blue) was for nucleus staining. Profile line fluorescence curves were generated using Zen 2010 software (Right). Scale bar 10 μm. (C) ELISA detection of IL-6 release level in the supernatant. The data are presented as the mean ± standard deviation. * $p < 0.05$, compared with the Ctrl group. # $p < 0.05$, compared with the ox-LDL+PGE2 group.

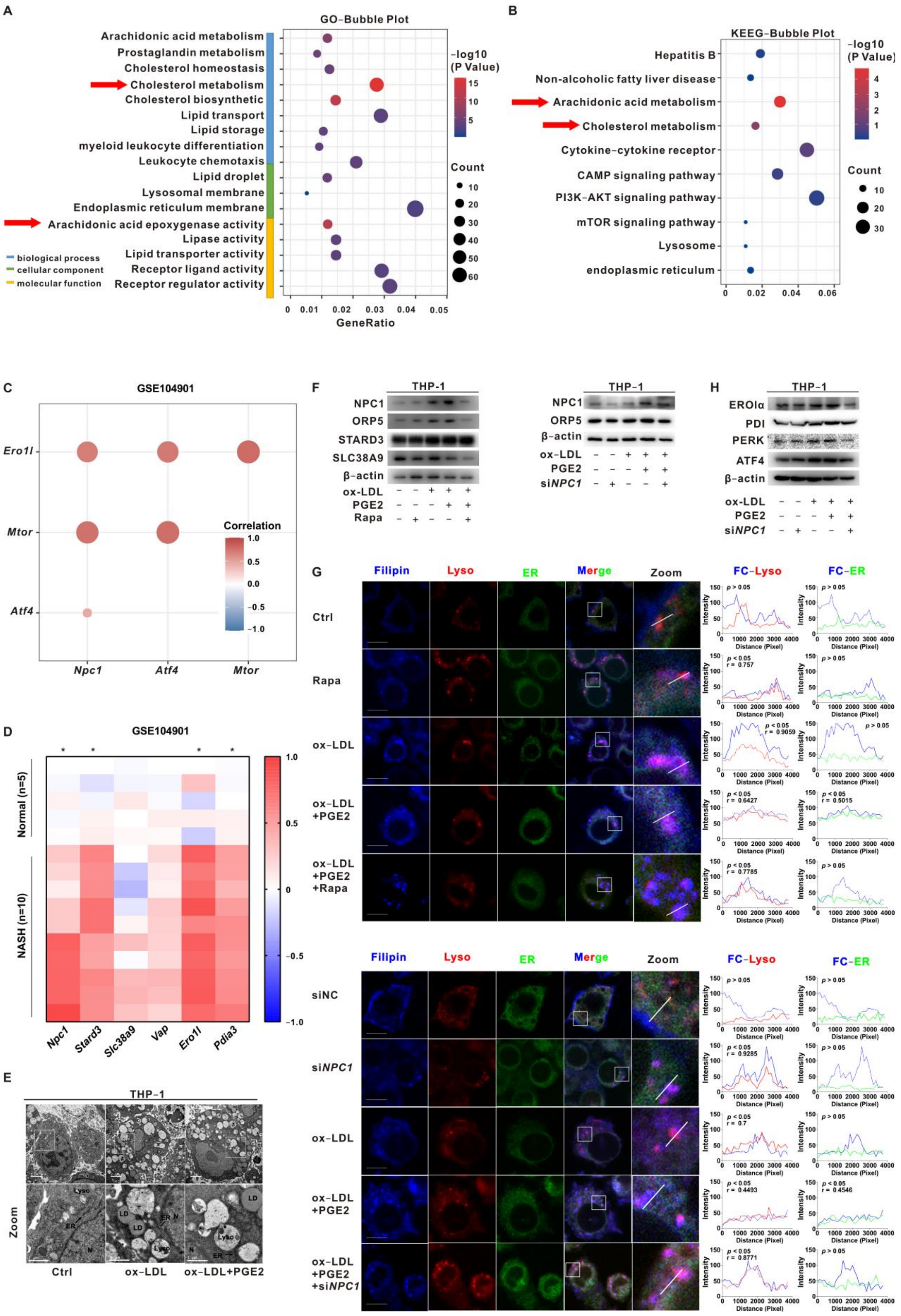

Figure S3. NPC1 mediated Lyso-ER cholesterol transport to regulate ER redox and ER stress homeostasis. (A, B) Transcriptomics analysis of HBx-Tg mice liver. Arrows indicate the cholesterol- and PGE2-related pathways screened in this study. (A) GO enrichment was displayed for analysis of DEGs. (B) KEGG enrichment was displayed for analysis of DEGs. (C, D) GEO database (accession no.: GSE104901) was

screened to analyze. (C) Pearson's correlation analysis was used for the relationship between mRNA levels of *Mtor*, *Atf4*, *Npc1*, and *Ero1l*. (D)The heatmap of Lyso-ER cholesterol transport and ER redox-related genes in liver macrophages from the NASH mice (n = 10) and the normal mice (n = 5). * $p < 0.05$, compared with the normal group. (E) THP-1 cells were analyzed for the lysosome and endoplasmic reticulum distribution by TEM. N: nucleus, Lyso: lysosome, ER: endoplasmic reticulum, LD: lipid droplet. Scale bars: 2 μm (Upper) and 1 μm (Lower). (F) WB detection of lysosomal cholesterol transport-related protein levels. (G) Representative images for evaluating the level of free cholesterol (blue), lysosome (red), ER (green), the colocalization (purple) between free cholesterol and lysosome, and the colocalization (cyan) between free cholesterol and ER (Left). Profile line fluorescence curves were generated using Zen 2010 software (Right). Scale bar 10 μm. (H) WB detection of ERO1α, PDI, and PERK.

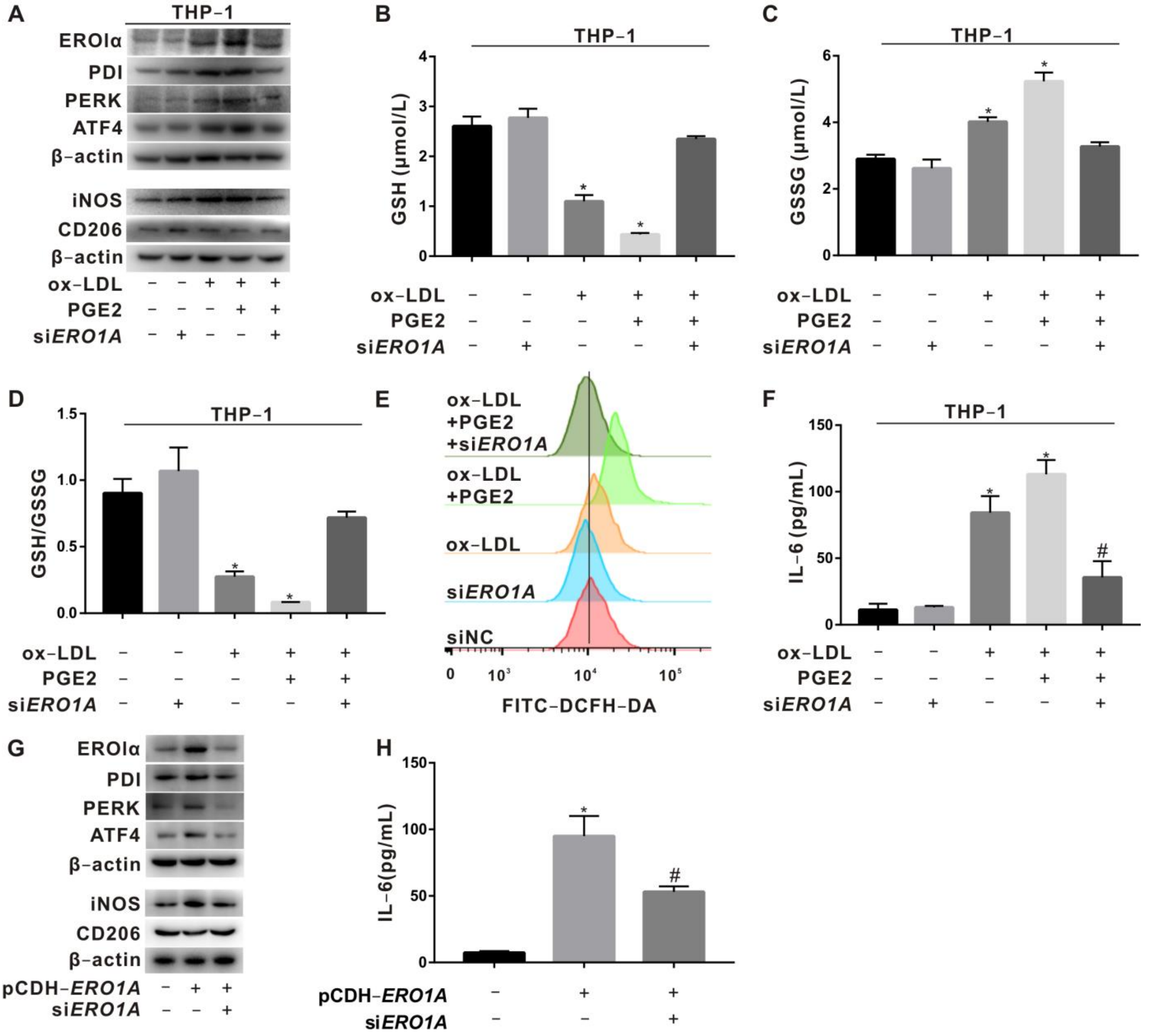

Figure S4. The targeted intervention effect of si*ERO1A*. (A) WB detection of ER redox, ER stress, and polarization marker protein levels. (B-D) Detection of reduced glutathione (GSH) and oxidized glutathione (GSSG) levels. (E) Flow cytometry detection of total ROS levels. (F) ELISA detection of IL-6 release level in the supernatant. (G) Detection of ER redox, ER stress, and polarization markers proteins in

THP-1-pCDH-*ERO1A* cells. (H) ELISA detection of IL-6 release level in the supernatant. The data are presented as the mean ± standard deviation. * $p < 0.05$, compared with the Ctrl group. # $p < 0.05$, compared with the ox-LDL+PGE2 or pCDH-*ERO1A* group.

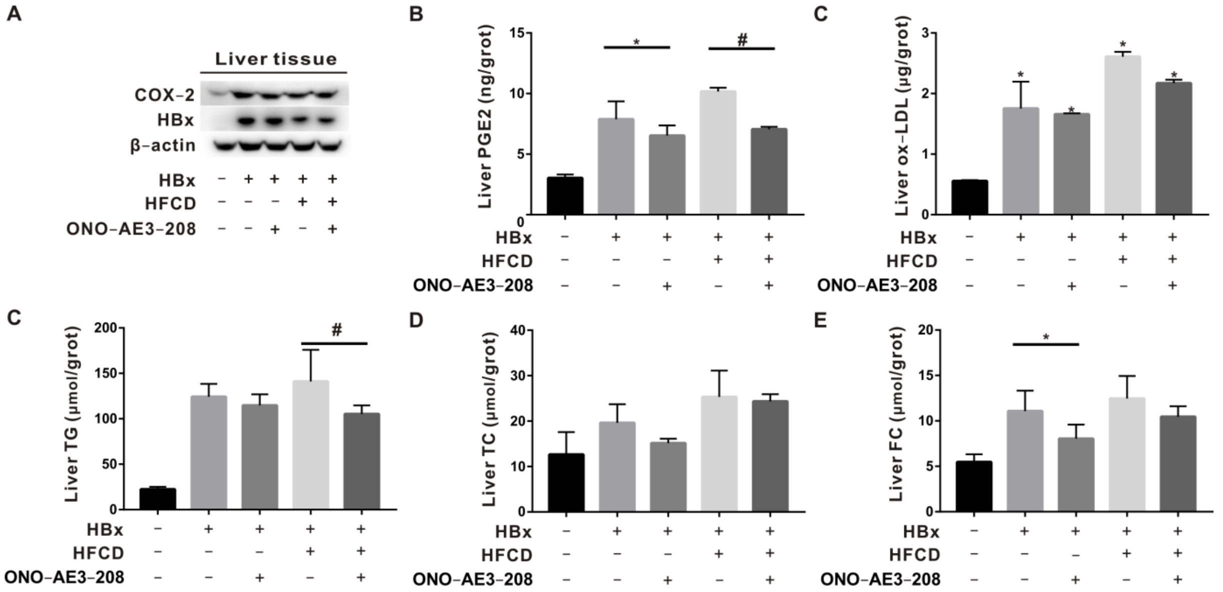

Figure S5. Assays for evaluating EP4 intervention to rescue HBx-associated NASH in vivo. C57BL/6-HBx-Tg (transgenic) mice and wildtype (WT) mice were used to establish the related models fed with high-fat/cholesterol diet (HFCD) or not, and administered with ONO-AE3-208 (5 mg/kg, intraperitoneal injection, once every other day for 2 weeks) for EP4 intervention. (A) WB detection of HBx, COX-2 in liver tissues. (B, C) ELISA detection of PGE2 and ox-LDL levels in the livers. (C-E) Detection of TG, TC, and FC level in the livers. The data are presented as the mean ± standard deviation. * $p < 0.05$, compared with the HBx group. # $p < 0.05$, compared with the HBx + HFCD group.

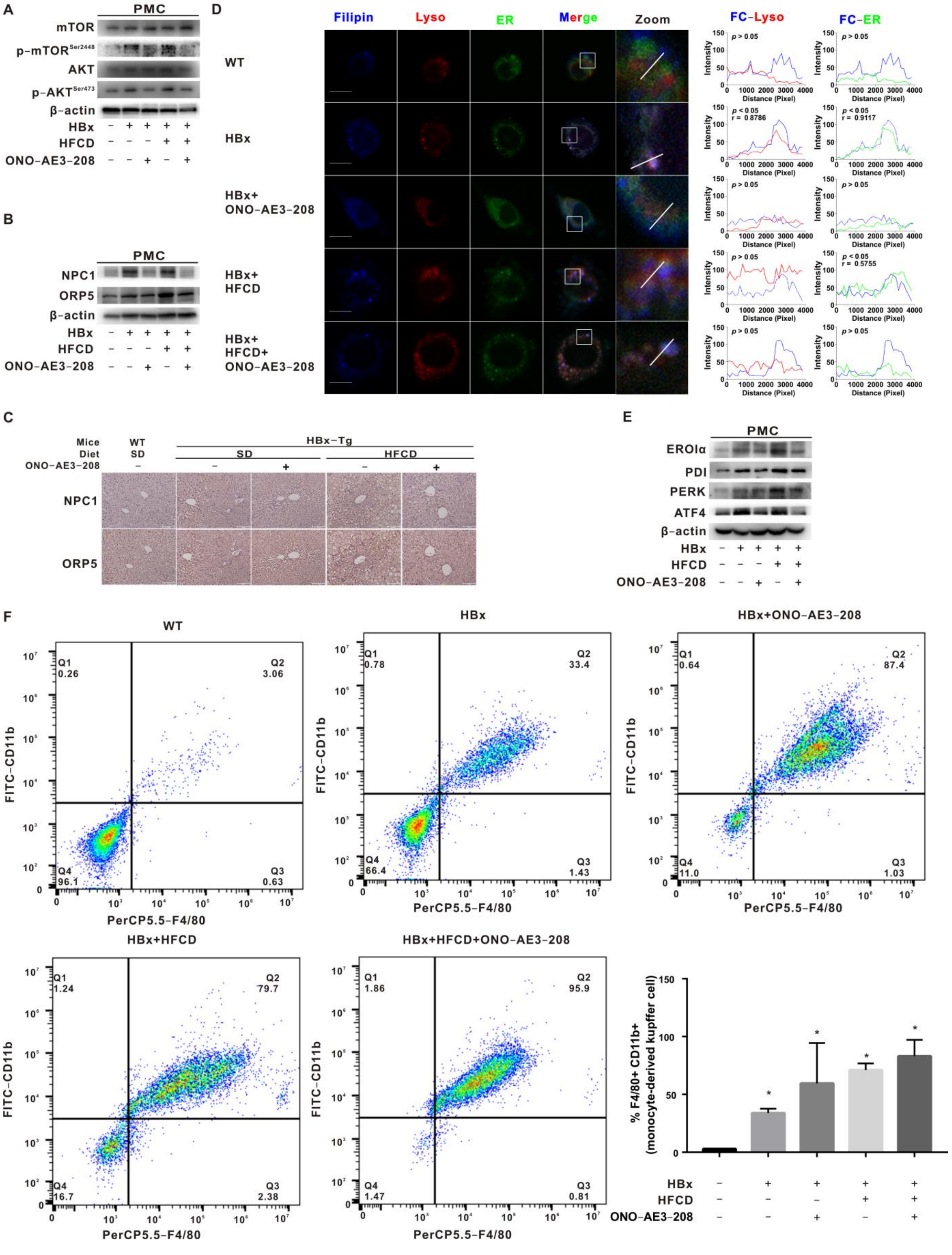

Figure S6. Assays for evaluating EP4 intervention to rescue hepatic macrophage polarization imbalance in vivo. (A, B) Primary macrophages (PMCs) were isolated by liver perfusion from different HBx-Tg mice models. (A) WB detection of AKT-mTOR signal protein levels in PMC. (B) WB detection of NPC1 and ORP5 protein levels in PMC. (C) Representative images of IHC staining of NPC1 and ORP5 protein levels in liver tissues. Scale bar 100 μm. (D) Representative images for evaluating the level of free cholesterol

(blue), lysosome (red), ER (green), the colocalization (purple) between free cholesterol and lysosome, and the colocalization (cyan) between free cholesterol and ER (Left). Profile line fluorescence curves were generated using Zen 2010 software (Right). Scale bar 10 μm. (E) WB detection of ERO1α, PDI, and PERK protein levels in PMC. (F) Flow cytometry of the proportion of monocyte-derived Kupffer cells in macrophages of mouse livers. The data are presented as the mean ± standard deviation. * $p < 0.05$, compared with the WT group.
